# Bronchoesophageal fistula: a rare complication of aortic endograft infection

**DOI:** 10.1259/bjrcr.20170061

**Published:** 2017-11-03

**Authors:** Russell Mark Salamo, Michael Moritz, Nadeem Parkar

**Affiliations:** Department of Radiology, Saint Louis University School of Medicine, St. Louis, MO, USA

## Abstract

Endovascular aortic aneurysm repair is an increasingly common approach for aortic aneurysm repair. Infection of the prosthetic is a rare, but devastating complication which may result in the well-known aortoenteric or aortobronchial fistulae. Bronchoesophageal fistula resulting from an infected aortic endograft has not yet been reported in the literature. Early recognition of the symptoms and prompt imaging confirmation are essential for treating an otherwise highly morbid diagnosis.

## Clinical presentation

A 66-year-old Caucasian male 11 days status post-thoracic endovascular aortic repair (TEVAR) for a ruptured thoracic aortic aneurysm presented to the emergency department complaining of chest pain and haemoptysis. Past medical history was significant for chronic gastritis, hypertension and coronary artery disease complicated by myocardial infarction and ischaemic cardiomyopathy. Patient was a current 20-pack year smoker with rare alcohol use.

## Investigations

**Figure 1.**
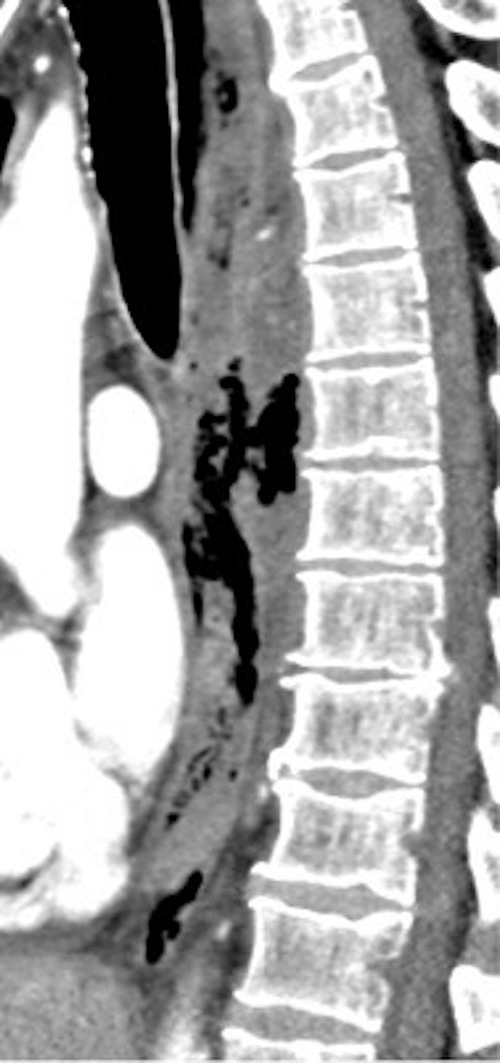
Sagittal CECT demonstrating a large amount of pneumomediastinum in the middle mediastinum with apparent discontinuity of the posterior esophageal wall. CECT, contrast-enhanced CT.

**Figure 2.**
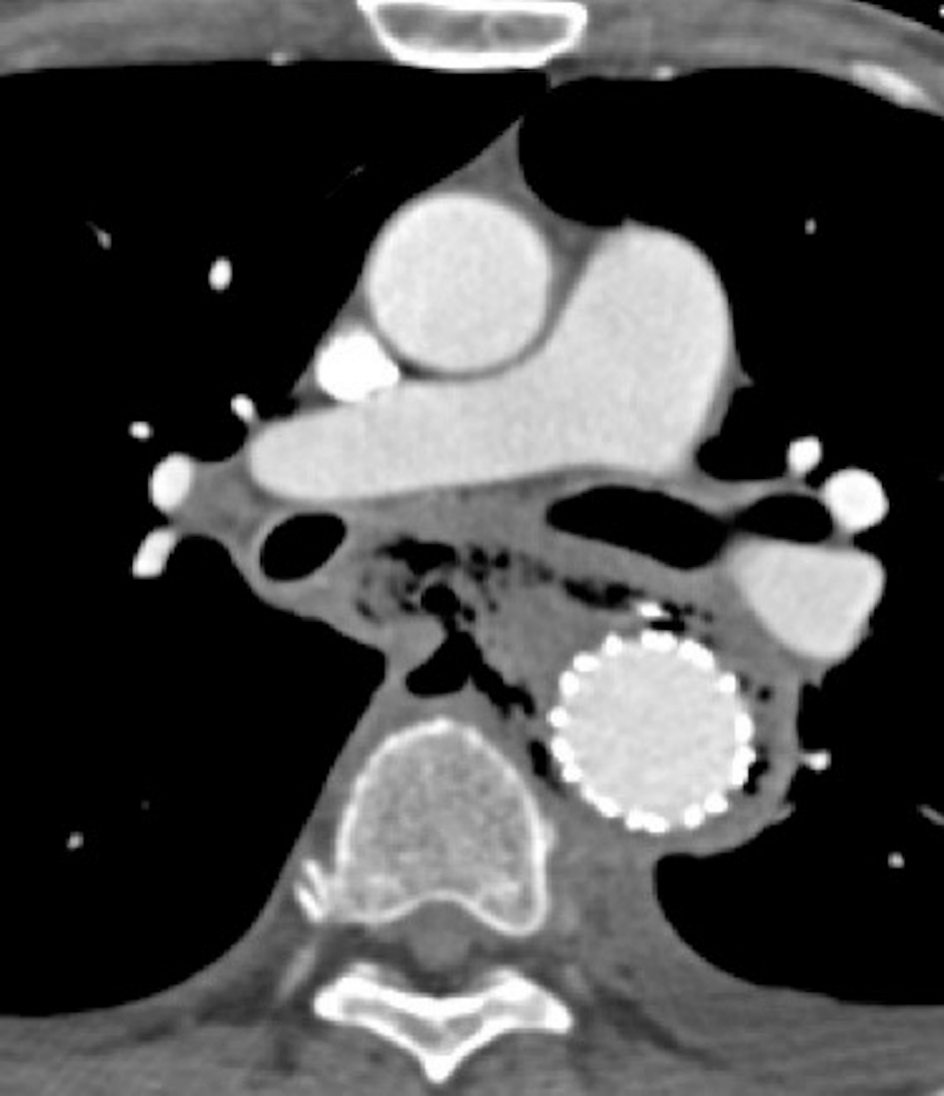
Axial CECT also showing large pneumomediastinum within the middle and posterior mediastinum surrounding the endovascular graft, with discontinuity of the posterolateral esophageal wall. CECT, contrast-enhanced CT.

To evaluate the graft, a contrast-enhanced CT (CECT) of the chest was performed according to a post-TEVAR protocol (at the author’s institution this includes non-contrast, angiographic, and delayed phases). The examination revealed extensive pneumomediastinum centred around the endovascular stent, involving the excluded aneurysm sac and extending into the middle mediastinum surrounding the esophagus and trachea. At this level, there was a gross discontinuity in the posterolateral esophageal wall measuring approximately 2 cm transverse by 3 cm craniocaudal (Figures 1 and 2).

**Figure 3.**
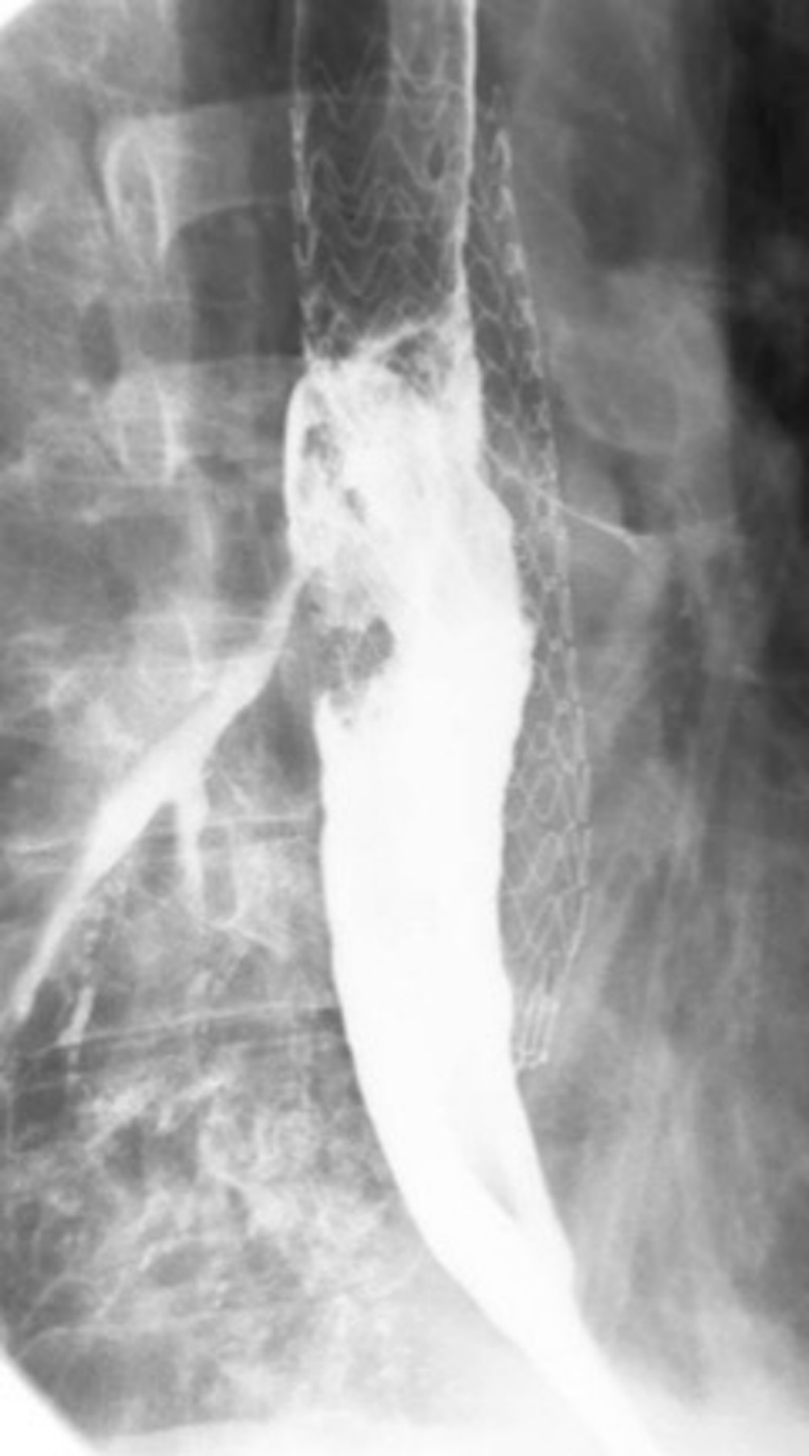
Fluoroscopic single contrast esophagram shows the contrast medium exiting the mid-thoracic esophagus and entering the right lower lobe bronchial tree.

Given the apparent esophageal wall abnormality, a single contrast fluoroscopic esophagram (Figure 3) was obtained utilizing water-soluble contrast in the anteroposterior and oblique projections which demonstrated contrast exiting the esophagus at the level of the mid TEVAR graft and entering the right lower lobe bronchial tree, consistent with a bronchoesophageal fistula (BEF).

## Differential diagnosis

The differential diagnosis for a patient presenting with haemoptysis in the early post-op period following TEVAR should include endoleaks, aortoenteric fistula and aortobronchial fistula.^1,2^ CECT of the chest was done to evaluate the integrity of the graft and surrounding structures, as well as to identify common non-TEVAR related causes of symptoms including but not limited to pneumonia and pulmonary embolus. In this case, the extensive pneumomediastinum surrounding the graft was suspicious for perigraft infection. The apparent discontinuity in the esophagus suggested an esophageal perforation; however, only after the fluoroscopic esophagram was a bronchoesophageal fistula considered.

## Treatment

Following confirmation of the BEF and given the suspicion for extensive perigraft infection, the patient was taken for angiography which again did not demonstrate an endoleak or aortoenteric fistula. On the following day, he underwent a right thoracotomy, esophagectomy with primary repair of the BEF, drainage of a mediastinal abscess, and reconstruction with intercostal and serratus muscle flaps.

## Outcome and follow-up

**Figure 4.**
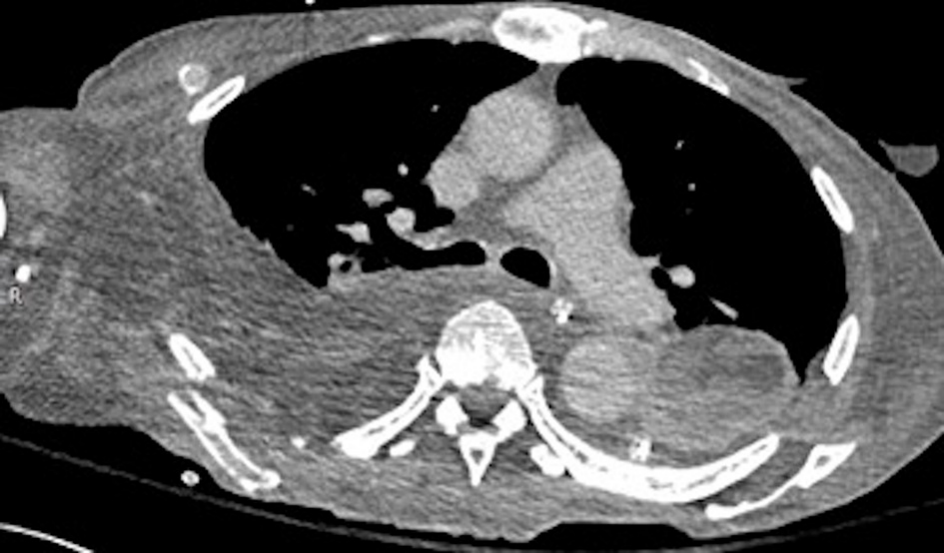
Axial CECT showing post-operative changes following repair of the fistula, evacuation of the mediastinal abscess, and serratus flap reconstruction.

Post-operatively, the patient was continued on broad-spectrum antibiotics including intravenous vancomycin, cefepime, metronidazole, and fluconazole. 3 days later, a bronchopleural fistula was discovered, which required repair with a pericardial patch and serratus muscle flap (Figure 4). Following a long recovery, the patient was discharged home.

## Discussion

TEVAR has emerged as the preferred approach for aortic aneurysm repair in many cases.^3,4^ Infection of the endograft is a rare, but often devastating complication, reportedly occurring in 0.6 to 3% of abdominal and thoracic aortic endovascular repairs.^5,6^

BEF is an abnormal communication between the esophagus and the bronchial tree. Symptomatic BEF often presents with bouts of cough and choking after eating, known as Ono’s sign, that is worse with liquids compared to solids.^7^ The haemoptysis seen in this patient was likely the result of lung suppuration that resulted from the endograft infection.^8^ BEF is rare in the adult and can be either acquired or congenital.^9,10^ Commonly encountered causes of BEF in adults include malignancy (most commonly esophageal or tracheobronchial in origin), trauma, and as a complication of infection (typically from chronic granulomatous infections such as tuberculosis, histoplasmosis and syphilis via abscess formation or from the erosion of adjacent structures by infected lymph nodes).^11,12^ BEF due to bronchial dehiscence has also been reported as a complication following lung transplantation.^13^ BEF resulting from an infected aortic endograft has not previously been reported in the literature.

Potential complications of TEVAR are typically first evaluated by CECT of the chest, which can also identify many common non-graft-related causes of symptoms. If there is concern for a BEF based on symptoms or prior imaging, patients should undergo a fluoroscopic esophagram, the best test for demonstrating the fistula tract with a capability of sensitive diagnosis in 78% of cases.^10^ Endoscopy and/or bronchoscopy may also be used to visualize the fistula opening and, in some situations, can be used to seal the fistula tract. CT esophagography has been proposed as an alternative to the conventional fluoroscopic esophagram in cases of suspected perforation, with the benefit of better delineating the extent of the perforation and, in this scenario, potentially reducing time to treatment if performed at the time of CTA. While CT esophagraphy, in the author’s experience, has proven useful in the setting of suspected esophageal injury, well-controlled studies comparing the two examinations have yet to be performed.^14,15^

Though less common than the other complications of endograft infection, radiologists should be aware of this diagnosis.

## Learning points

1. In adults, the most common etiology of broncho esophageal fistula (BEF) is malignancy (most commonly esophageal or tracheobronchial in origin), trauma, and as a complication of chronic granulomatous infections.
2. Infection of the endograft is a rare, but often devastating complication of endovascular aortic repair, reportedly occurring in 0.6 to 3% of abdominal and thoracic aortic endovascular repairs
3. Symptomatic BEF often presents clinically as Ono’s sign (coughing and choking after eating).
4. While CT is critical for diagnosing the etiology of the BEF, fluoroscopic esophagram is the best imaging modality for visualizing the fistula itself.

## Consent

Written informed consent was obtained from the patient for publication of this case report, including the accompanying images.
